# Antibiotic-Related Adverse Drug Reactions in Patients Treated on the Dermatology Ward of Medical University of Gdańsk

**DOI:** 10.3390/antibiotics10101144

**Published:** 2021-09-22

**Authors:** Ewa Maria Sokolewicz, Martyna Rogowska, Miłosz Lewandowski, Monika Puchowska, Dorota Piechota, Wioletta Barańska-Rybak

**Affiliations:** 1 Department of Dermatology, Venerology and Allergology, Medical University of Gdańsk, Debinki 7 Street, 80-952 Gdańsk, Poland; martyna.rogowska@gumed.edu.pl (M.R.); milosz.lewandowski@gumed.edu.pl (M.L.); dr.dorota.piechota@gmail.com (D.P.); wioletta.baranska-rybak@gumed.edu.pl (W.B.-R.); 2 Department of Oncology and Radiotherapy, Medical University of Gdańsk, Debinki 7 Street, 90-952 Gdańsk, Poland; monika.puchowska@gumed.edu.pl

**Keywords:** adverse drug reactions, antibiotics, beta lactams

## Abstract

Adverse drug reactions (ADRs) are unexpected reactions to a medication administered in a correct way at a standard dose. Drug-induced skin reactions account for 60–70% of all ADRs. The aim of the study is to determine the prevalence of antibiotic-related dermatological ADR in patients treated in the department of Dermatology, Venerology and Allergology of the University Clinical Center in Gdańsk, Poland, in the years 2004–2021. A retrospective analysis of patients’ medical files was conducted in order to identify cases of ADR connected with the use of antibiotics, yielding 84 cases. The most common group of antibiotics were β-lactam, causing ADR in 47 patients. β-lactam antibiotics in our study included amoxicillin, alone and combined with clavulanic acid, and cephalosporins, affecting 22, 18 and 7 patients, respectively. In conclusion, β-lactam antibiotics showed the highest prevalence among antibiotic-induced skin reactions. They accounted for 15% of cases of all dermatological drug reactions and 55% of those caused by antibiotics. Especially amoxicillin, prescribed as a single drug or in combination with clavulanic acid, was commonly the culprit. Due to its wide use in the hospital and outpatient clinic, these adverse reactions have to be kept in mind by both hospital staff and general practitioners.

## 1. Introduction

Based on the definition created by the World Health Organization, an adverse drug reaction (ADR) is “a response to a drug which is noxious and unintended, and which occurs at doses normally used in man for the prophylaxis, diagnosis, or therapy of disease, or for the modifications of physiological function” [1]. While up to 80% of ADRs are considered predictable and dependent on the administrated dose, about 20% are dose-independent and unpredictable [2]. Cutaneous adverse drug reactions can be divided into those not posing a threat to the life of the affected individual and life-threatening reactions. The former manifest as a large variety of skin reactions such as rashes, including a morbilliform rash, urticaria, fixed drug eruption, purpura, or vasculitis [3]. However, certain individuals experience far more severe cutaneous adverse drug reactions (SCAR) than those aforementioned. The term SCAR encompasses cutaneous manifestations such as urticarial and maculopapular exanthema (MPE) and their more severe counterparts, namely, acute generalized exanthematous pustulosis (AGEP), a drug rash with eosinophilia and systemic symptoms (DRESS), Stevens–Johnson syndrome (SJS) and toxic epidermal necrolysis (TEN), the diagnosis of the latter two depending on the percentual extent of skin desquamation [4]. Not seldom the clinical picture is enough for physicians to establish the diagnosis of a cutaneous adverse drug reaction. Furthermore, it is often the patients themselves who link the occurrence of their skin reactions with the intake of a certain drug or change in their medication regimen, such as the addition of a new pharmacological agent or change of its dose. If not, however, a meticulously gathered patient history is often of help. Especially given the fact that, according to MA Riedl et al., the time frame in question when taking patient drug history should include a recording of all prescription and nonprescription drugs taken within the last month, including dates of administration and dosage. Excluding the case of a previous drug sensitization, “the interval between the initiation of therapy and the onset of reaction is rarely less than one week or more than one month” [5]. Nonetheless, even with highly suggestive cutaneous presentations and a corresponding patient history, a dermatological consultation with a potential skin biopsy should be performed in order to establish a definite clinical diagnosis [6]. Out of all drug classes, antibiotics are considered the most common cause of life-threatening immune-mediated drug reactions that are considered off-target, including severe cutaneous adverse reactions [7]. Given the public health importance of antibiotics and the high frequency of antibiotic intake in society, we decided to analyze the prevalence of antibiotic-related dermatological adverse drug reactions in patients treated in the department of Dermatology, Venerology and Allergology of the University Clinical Center in Gdańsk, Poland, in the years 2004–2021.

## 2. Results

Drug reactions were reported in 84 patients, 65% of whom were women and 35% were men. Among the antibiotics, the β-lactam group was distinguished, including amoxicillin, cephalosporins and amoxicillin–clavulanic acid. These three antibiotics were responsible for 56% of all drug-related reactions.

**Figure 1:**
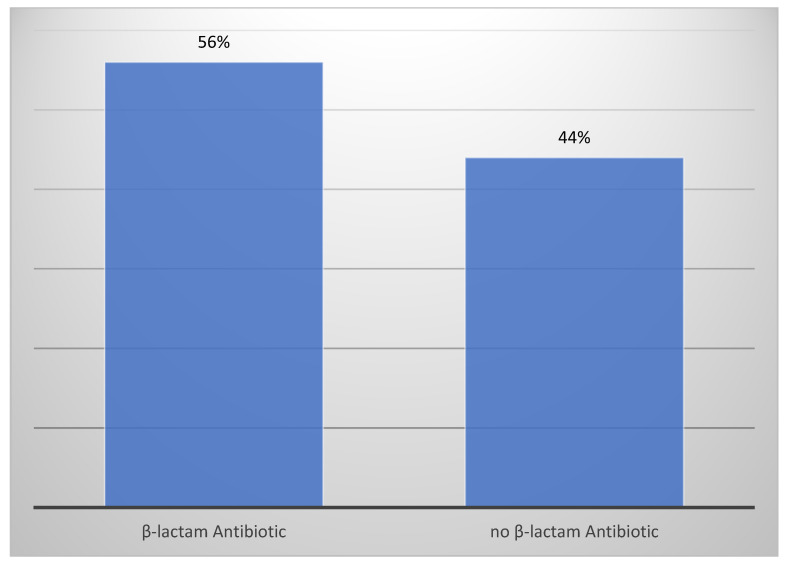
Percentage of β-lactams partaking in cutaneous drug reactions after exposure to antibiotics.

**Figure 2:**
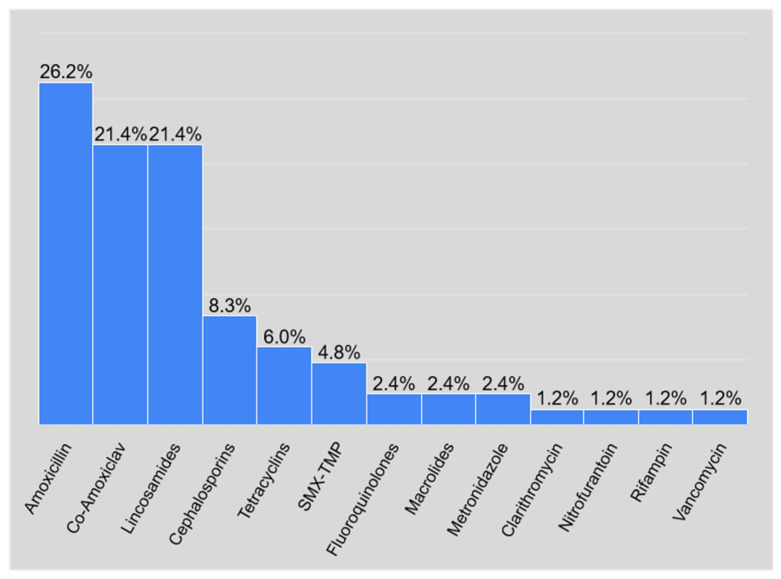
Percentage of cutaneous drug reactions depending on the type of antibiotic being the causative agent.

Among all admissions of patients to the department of Dermatology, Venerology and Allergology of the University Clinical Center in Gdańsk, Poland, in the years 2004–2021, 84 cases were linked to the use of drugs classified as antibiotics. Overall, the following antibiotics were determined to be causative agents in our set of patients: the group of penicillins, represented by amoxicillin prescribed as a sole agent or combined with clavulanic acid, cephalosporins of 2nd and 3rd generation, cefuroxime and ceftriaxone, respectively, the group of lincosamides, including agents such as lincomycin or clindamycin and tetracyclines. Furthermore, folic acid antagonists in the form of trimethoprim/sulfamethoxazole, as well as macrolides, were determined. Fluoroquinolones, metronidazole, vancomycin, nitrofurantoin and rifampin were also found to be causative agents in several patients. The most common group of antibiotics was β-lactams, causing drug-induced skin reactions in 47 patients. β-lactam antibiotics in our study included amoxicillin, alone and combined with clavulanic acid, and cephalosporins, affecting 22, 18 and 7 patients, respectively. 56% of reactions were caused by β-lactam, in comparison to the remaining 44% of cutaneous manifestations after exposure to antibiotics from another group, which has been demonstrated in Figure 1. Another significant group in our research was lincosamides, being the culprit in 18 cases. Five cases of dermatological ADR after tetracyclines were identified. Folic acid antagonists affected four patients and macrolides two patients. Only two cases each were linked to fluoroquinolones and metronidazole. Vancomycin, nitrofurantoin and rifampin were associated with the smallest amount of cases, each affecting one patient. Figure 2 constitutes the visual representation of the aforementioned data.

The drugs that showed the most common side effect were:

- Amoxicillin (22 cases, 26.2% of all reported cases).
- Amoxicillin–clavulanic acid and lincosamides (18 cases each, which accounted for 21.4% of all reported cases).

**Figure 3:**
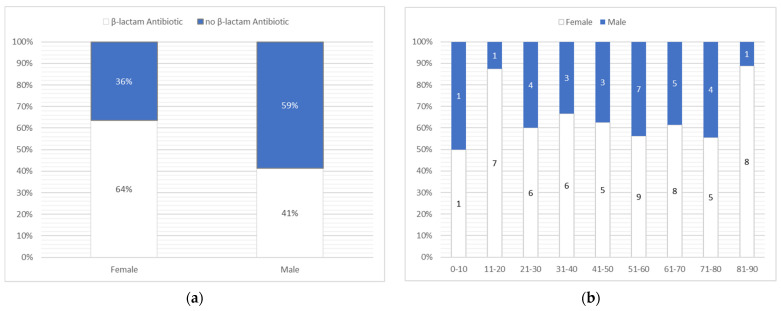
Analysis of the group based on gender. (**a**) Correlation between and the type of antibiotic group causing cutaneous adverse drug reaction; (**b**) sex distribution based on age.

There was a slight difference between the sexes and the incidence of drug reactions in relation to the group of antibiotics. Women experienced more reactions after taking β-lactam antibiotics than men (Figure 3a). As Figure 3b shows, after dividing our group of patients based on their gender, the biggest inequality was observed among patients between ages 81–90, where 8 female patients were affected and only one man. As seen in Table 1, the results could also be analyzed taking the cumulative number and percent under consideration. Dividing the cumulative frequency by the total number of observation yielded a cumulative percentage. In the literature, this value is also known as a running total of the percentage.

## 3. Discussion

The female gender surely plays a role in the probability of developing a drug-induced reaction. Several studies prove that according to their registry of patients hospitalized due to drug allergy, patients of the female gender were statistically significantly more likely to develop a drug allergy than males [8]. However, interestingly enough, a significant discrepancy in both, the clinical picture and mortality, could not be proven between the two gender groups [9]. Furthermore, a sensitivity based on ethnic background or localization should also be taken into consideration. According to Gerogianni K. et al., genetic associations vary between different ethnic populations [10]. For instance, a study was conducted in order to investigate the prevalence, incidence and sensitization profile of β-lactam antibiotic allergy in Hong Kong by Philip H. Li et al. The outcomes of this study were highly suggestive that patients in Hong Kong with β-lactam antibiotic allergy had much higher rates of monosensitization to benzylpenicilloyl polylysine and benzylpenicilloate, proving these reagents to be essential in β-lactam antibiotic skin tests. Furthermore, yielding the question, whether such sensitization is specific to ethnicity or region [11]. Additionally, if so, can drug allergies and drug-induced cutaneous skin reactions be predicted solely on the ethnic profile of the population in the region? So far, various algorithms have been created in order to evaluate adverse drug reactions, drug hypersensitivity and allergy. One of the most often referred to was created by the European Network for Drug Allergy (ENDA). The organization has created numerous diagnostic algorithms in order to facilitate the evaluation of immediate [12] and nonimmediate reactions [13]. An algorithm strictly based on adverse drug reactions expressed on the skin and correlated with patients’ ethnic background, however, might be of benefit.

A study from Silviu Dan F. et al., on the other hand, focused on the frequency of skin test reactions to side chain penicillin determinants. It showed that of 21 patients, the reactivity of a skin test was in 47.6% limited to semisynthetic penicillin reagents derived from ampicillin, amoxicillin or cloxacillin. Furthermore, in the study conducted, it was ampicillin and amoxicillin which were the semisynthetic p-lactams responsible for the biggest quantity of clinical reactions—ampicillin with 24.1% and amoxicillin being responsible for 33.9% of reactions. Additionally, the same study also showed that ampicillin was, apart from derivatives of benzylpenicillin, the most common derivative of penicillin in which skin tests noted a reactivity in 38.1% of cases. In comparison, a 52.3% of skin test reactivity was noted with benzylpenicillin derivatives [14]. In our study, β-lactam antibiotics denoted the vast majority of drug-induced skin reactions, which was consistent with other studies. In fact, when analyzing adverse drug reactions with an underlying immunologic mechanism, β-lactams are the most important culprits according to Dewdney J.M. Due to their frequent involvement and wide distribution, they are also the antibiotics considered the most thoroughly researched [15]. This can be directly or indirectly connected to their accessibility and high consumption [16].

A lot of research focuses on hypersensitivity reactions to β-lactam antibiotics in childhood. For example, Blanca, et al.’s article focused solely on the outcomes in the pediatric population [17]. In light of our median age of the patients and high age range of the group of patients we gathered, we believe the next step would be to thoroughly examine and research the pediatric population based on the antibiotic or group of antibiotics being the causative agents, the age at the time of the first manifestation of the symptoms and, also, the type of dermatological reaction connected with them.

Nonetheless, the misdiagnosis of a cutaneous adverse drug reaction, drug hypersensitivity or allergy can also be detrimental. Misdiagnosing an individual with a drug allergy yields to limiting their drug regimen and diminishing possible therapeutic options. As previously mentioned, there is a higher consumption of these drugs, they are widely distributed and easy for both physicians and patients to access. The results of a study by Nelson A. et al. allowed the conclusion that the incorrect diagnosis of penicillin allergy frequently leads to the exclusion of this drug as a therapeutic option. A better recognition of these situations will enable the use of penicillin and reduce the risks associated with hypersensitivity [18].

Diagnostics of cutaneous drug eruptions is hampered by the fact that one drug may induce different eruptions while the same cutaneous eruption can be caused by several drugs [19]. An option worth considering is implementing an algorithm encompassing the performance of a clinical or laboratory assay into daily practice, in order to correctly assess a possible cutaneous adverse drug reaction. Goldberg, et al. analyzed a large variety of tests, including the radioallergosorbent test (RAST), mast cell degranulation test, lymphocyte transformation test, lymphocyte toxicity assay, as well as the test for the diagnosis of reactions that involve immune complexes, the macrophage migratory inhibition factor test and interferon-gamma release test [20]. Research by Halevy S et al. also suggests that the in vitro drug-induced IFN-gamma release test may serve as a diagnostic tool in cutaneous adverse drug reactions [21], not only to diagnose the reaction itself, but also to aid finding the pharmacological culprit [22].

Posing an incorrect diagnosis of penicillin or β-lactam allergy also carries another risk. It yields shifting the pharmacologic therapy to cephalosporins. However, there are increasing amounts of reports proving that this group of antibiotics also causes a significant amount of adverse drug reactions. As stated by the review by ME Pichichiero, who retrieved 219 articles out of which 106 served as source material, a significant increase in allergic reactions to cephalothin, cephaloridine, cephalexin, cefazolin and cefamandole was observed in patients considered to be penicillin-allergic. According to his work, no increase was observed with cefprozil, cefuroxime, ceftazidime or ceftriaxone. The cross-allergy between penicillins and cephalosporins is attributed to similarities in side chains. In order to establish a prediction of a cross-allergy, clinical challenges, skin testing and studies based on monoclonal antibodies can be taken under consideration [23].

In our study, a considerable amount of dermatological manifestations of adverse drug reactions due to antibiotics were linked to lincosamides. Lincosamides include two important agents, lincomycin and clindamycin [24]. As antibiotics, they bind to the 23S RNA and block the activity of the bacterial ribosome, effectively inhibiting protein synthesis [25]. Clindamycin is usually much more active than lincomycin in the treatment of bacterial infections, in particular those caused by anaerobic species [26]. Numerous studies, such as the one proving their effectiveness also in dermatology by treating uncomplicated skin infections such as cellulitis or abscesses [27]. However, there are increasing case reports on dermatological manifestations of ADR due to lincosamides. For instance, acute generalized exanthematous pustulosis due to clindamycin [28]. Acute generalized exanthematous pustulosis (AGEP) is a severe pustular cutaneous adverse drug reaction. It is a reaction characterized by sterile, non-follicular pustules overlying the erythematous skin [29]. It was previously associated mainly with the intake of β-lactam and macrolide antibiotics [30,31]. Recently, the manifestations of AGEP have been increasingly connected to lincosamide antibiotics as well, particularly clindamycin [32]. Furthermore, the article by De Cruz R et al. reporting a localized acute exanthematous pustulosis (ALEP) induced by clindamycin in pregnancy [33] is a reminder that proves not only that similar to many skin reactions exanthematous pustulosis can be generalized or local, but also the importance and cautiousness needed in managing a pregnant patient and prescribing antibiotics to them. Antibiotics can not only cause adverse cutaneous drug reactions in this set of patients, but also carry a wide variety of risks of malformations or pyloric stenosis or the risk of a spontaneous abortion [34,35].

## 4. Materials and Methods

Retrospective analysis of medical files of 84 patients experiencing dermatological adverse drug reactions connected with the use of antibiotics was conducted in order to thoroughly analyze them.

We investigated cases of patients admitted to the ward in the years 2004–2021. The amount of patients was different each year. Between 2004 and 2010, there was a necessity to find data in the hospital archives. After the year 2010, it was possible to find information about the patients in the hospital’s online system.

All the patients experiencing adverse drug reactions during this period of time were divided into smaller subgroups based on the pharmacological groups of causative medication. This study focused on our findings connected solely with the intake of antibiotics.

A table was created in order to analyze the researched group based on the patient’s age, gender and the antibiotic which was stated to be the culprit.

The entire research based on the findings is listed in aforementioned Table and encompassed solely the patients included in the stated Table.

In order to verify the researched data, statistical analyzes were carried out using the Statistica 13 package. Descriptive statistics were analyzed with its use.

## 5. Conclusions

Despite the study being quite preliminary in the present form and its retrospective character, the following conclusions could be determined. β-lactam antibiotics showed the highest prevalence among antibiotic-induced skin reactions. They accounted for 15% of cases of all dermatological drug reactions and 55% of those caused by antibiotics. Especially amoxicillin, prescribed as a single drug or in combination with clavulanic acid, can be the culprit. Due to its wide use in the hospital as well as in the outpatient clinic, these adverse reactions have to be kept in mind by both hospital staff and general practitioners. Furthermore, cutaneous adverse drug reactions connected with the intake of lincosamides must also not be forgotten, as those pharmacological agents are commonly used, but, simultaneously, also account for a large number of adverse drug reactions manifesting dermatologically.

## Figures and Tables

**Table 1:** The prevalence of drug reactions depending on the antibiotic.

Class	Number	Cumulative Number	Percent	Cumulative Percent
Amoxicillin	22	22	26.19048	26.1905
Co-Amoxiclav	18	47	21.42857	55.9524
Lincosamides	18	68	21.42857	80.9524
Cephalosporins	7	29	8.33333	34.5238
Tetracyclines	5	83	5.95238	98.8095
SMX-TMP	4	78	4.76190	92.8571
Fluoroquinolones	2	49	2.38095	58.3333
Macrolides	2	70	2.38095	83.3333
Metronidazole	2	72	2.38095	85.7143
Clarithromycin	1	50	1.19048	59.5238
Nitrofurantoin	1	73	1.19048	86.9048
Rifampin	1	74	1.19048	88.0952
Vancomycin	1	84	1.19048	100.0000

The drugs that showed the most common side effect were amoxicillin, accounting for 22 cases, 26.2% of all reported cases, and co-amoxiclav and lincosamides, each of the latter two responsible for 18 cases, which accounted for 21.4% of all reported cases. Women experienced more reactions after the intake of β-lactam antibiotics. Age-wise, the group was quite broad, ranging from 2 to 89 years. The mean age of the patients was 50.79 with a median of 53.5 years.

## References

[1] World Health Organization (1972). International Drug Monitoring: The role of National Centres. Report of a WHO Meeting.

[2] Nayak S., Acharjya B. (2008). Adverse cutaneous drug reaction. Indian J. Dermatol..

[3] Mockenhaupt M. (2017). Epidemiology of cutaneous adverse drug reactions. Allergol. Sel..

[4] Chang C.J., Chen C.B., Hung S.I., Ji C., Chung W.H. (2020). Pharmacogenetic Testing for Prevention of Severe Cutaneous Adverse Drug Reactions. Front. Pharmacol..

[5] Riedl M.A., Casillas A.M. (2003). Adverse drug reactions: Types and treatment options. Am. Fam. Physician.

[6] Lützow-Holm C., Rønnevig J.R. (2005). Kutane legemiddelreaksjoner [Cutaneous drug reactions]. Tidsskr Nor Laegeforen..

[7] Blumenthal K.G., Peter J.G., Trubiano J.A., Phillips E.J. (2019). Antibiotic allergy. Lancet.

[8] Thong B.Y., Tan T.C. (2011). Epidemiology and risk factors for drug allergy. Br. J. Clin. Pharmacol..

[9] Leong K.P., Thong B.Y., Cheng Y.K., Tang C.Y., Chng H.H. (2003). Are there differences in drug allergy between the sexes?. Ann. Acad. Med. Singap..

[10] Gerogianni K., Tsezou A., Dimas K. (2018). Drug-Induced Skin Adverse Reactions: The Role of Pharmacogenomics in Their Prevention. Mol. Diagn..

[11] Li P.H., Yeung H.H.F., Lau C.S., Au E.Y.L. (2020). Prevalence, Incidence, and Sensitization Profile of β-lactam Antibiotic Allergy in Hong Kong. JAMA Netw. Open..

[12] Torres M.J., Blanca M., Fernandez J., Romano A., Weck A., Aberer W., Brockow K., Pichler W.J., Demoly P., ENDA (2003). Diagnosis of immediate allergic reactions to beta-lactam antibiotics. Allergy.

[13] Romano A., Blanca M., Torres M.J., Bircher A., Aberer W., Brockow K., Pichler W.J., Demoly P., ENDA, EAACI (2004). Diagnosis of nonimmediate reactions to beta-lactam antibiotics. Allergy.

[14] Silviu Dan F., Mc Philips S., Warrington R. (1993). The frequency of skin test reactions to side chain penicillin determinants. J. Allergy Clin. Immunol..

[15] Weiss MAdkinson N.F. (1988). Immediate hypersensitivity reactions to penicillin and related antibiotics. Clin. Allergy.

[16] Blanca M. (1995). Allergic reactions to penicillins. A changing world?. Allergy.

[17] Blanca M., Torres M.J. (2003). Reacciones de hipersensibilidad a antibióticos betalactámicos en la infancia [Hypersensitivity reactions to beta-lactam antibiotics in childhood]. Allergol. Immunopathol..

[18] Rosário N.A., Grumach A.S. (2006). Allergy to beta-lactams in pediatrics: A practical approach. J. Pediatr..

[19] Mulder W.M., Meinardi M.M., Bruynzeel D.P. (2004). Huidreacties door geneesmiddelen [Cutaneous reactions to drugs]. Ned. Tijdschr. Geneeskd..

[20] Goldberg I., Gilburd B., Shovman O., Brenner S. (2004). Clinical and laboratory assays in the diagnosis of cutaneous adverse drug reactions. Isr. Med. Assoc. J..

[21] Halevy S., Cohen A.D., Grossman N. (2001). [In vitro interferon-gamma release--a laboratory diagnosis of cutaneous adverse drug reactions]. Harefuah.

[22] Halevy S., Cohen A.D., Grossman N. (2005). Clinical implications of in vitro drug-induced interferon gamma release from peripheral blood lymphocytes in cutaneous adverse drug reactions. J. Am. Acad. Dermatol..

[23] Pichichero M.E. (2007). Use of selected cephalosporins in penicillin-allergic patients: A paradigm shift. Diagn. Microbiol. Infect. Dis..

[24] Verdier L., Bertho G., Gharbi-Benarous J., Girault J.P. (2000). Lincomycin and clindamycin conformations. A fragment shared by macrolides, ketolides and lincosamides determined from TRNOE ribosome-bound conformations. Bioorg Med. Chem..

[25] Kulczycka-Mierzejewska K., Trylska J., Sadlej J. (2012). Quantum mechanical studies of lincosamides. J. Mol. Model..

[26] Spížek J., Řezanka T. (2017). Lincosamides: Chemical structure, biosynthesis, mechanism of action, resistance, and applications. Biochem. Pharmacol..

[27] Miller L.G., Daum R.S., Creech C.B., Young D., Downing M.D., Eells S.J., Pettibone S., Hoagland R.J., Chambers H.F., DMID 07-0051 Team (2015). Clindamycin versus trimethoprim-sulfamethoxazole for uncomplicated skin infections. N. Engl. J. Med..

[28] Sulewski R.J., Blyumin M., Kerdel F.A. (2008). Acute generalized exanthematous pustulosis due to clindamycin. Derm. Online J..

[29] Aiempanakit K., Apinantriyo B. (2020). Clindamycin-induced acute generalized exanthematous pustulosis: A case report. Medicine.

[30] Schwab R.A., Vogel P.S., Warschaw K.E. (2000). Clindamycin-induced acute generalized exanthematous pustulosis. Cutis.

[31] Rajgopal Bala H., Jalilian C., Goh M.S., Williams R., Tan G., Chong A.H. (2017). Two cases of amoxycillin-induced follicular acute localised exanthematous pustulosis. Australas. J. Dermatol..

[32] Smeets T.J., Jessurun N., Härmark L., Kardaun S.H. (2016). Clindamycin-induced acute generalised exanthematous pustulosis: Five cases and a review of the literature. Neth. J. Med..

[33] De Cruz R., Ferguson J., Wee J.S., Akhras V. (2015). Acute localised exanthematous pustulosis (ALEP) induced by clindamycin in pregnancy. Australas. J. Dermatol..

[34] Nordeng S., Nordeng H., Høye S. (2016). Bruk av antibiotika i svangerskapet [Use of antibiotics during pregnancy]. Tidsskr Nor Laegeforen..

[35] Muanda F.T., Sheehy O., Bérard A. (2017). Use of antibiotics during pregnancy and risk of spontaneous abortion. CMAJ.

